# Pressure support versus continuous positive airway pressure for predicting successful liberation from invasive ventilation in children: an open label, randomized non-inferiority trial

**DOI:** 10.1016/j.lansea.2023.100219

**Published:** 2023-05-29

**Authors:** C. Revaiah Vishwa, Karthi Nallasamy, Suresh Kumar Angurana, Arun Bansal, Muralidharan Jayashree

**Affiliations:** Pediatric Critical Care Unit, Advanced Pediatrics Centre, Postgraduate Institute of Medical Education and Research, Chandigarh, 160012, India

**Keywords:** Pressure support, CPAP, Spontaneous breathing trial, Extubation readiness test, Mechanical ventilation, PICU

## Abstract

**Background:**

Pressure support (PS) as a spontaneous breathing trial (SBT) was considered inferior to continuous positive airway pressure (CPAP) and T-piece because PS underestimated post-extubation work of breathing in physiologic studies. We aimed to compare PS and CPAP as SBT methods for assessing clinical outcomes in children.

**Methods:**

This was an open label randomized non-inferiority trial conducted between December 2019 and August 2021 among children aged 1 month to 12 years deemed ready for weaning after at least 48 h of invasive ventilation in PICU. Children were randomized to undergo a 2-h SBT with PS of 8 cm H_2_O in addition to PEEP 5–6 cm H_2_O or CPAP (5–6 cm H_2_O). The primary outcome was successful liberation from invasive ventilation for 72 h after first SBT. Secondary outcomes included first SBT pass rate, need for post-extubation respiratory support (high flow oxygen and/or non-invasive ventilation), and length of PICU stay.

**Findings:**

Of the 247 enrolled children, 244 completed the trial (121 in PS and 123 in CPAP group). Median (IQR) age was 24 (9, 84) months. Median (IQR) duration of invasive ventilation before randomization was 4.5 (3, 6.5) days. Successful liberation from invasive ventilation after first SBT occurred in 97 (80.2%) children in PS and 93 (75.6%) children in CPAP group [difference 4.6; 95% CI (−5.8, 15); p = 0.39]. First SBT pass rate between PS and CPAP [111 (91.7%) versus 105 (85.4%); difference 6.3; 95% CI (−1.6, 14.3); p = 0.12] was similar. Need for post-extubation respiratory support [52 (43%) versus 49 (40%)], rate of reintubation within 72 h [14 (11.6%) versus 12 (9.8%)] and median (IQR) length of PICU stay [9 (6, 15) versus 8 (5.5, 13) days] were comparable. Four (1.6%) children, all in CPAP group had unfavourable outcome (1 died, 3 discontinued care).

**Interpretation:**

In invasively ventilated children, 2-h SBT with pressure support was non-inferior to CPAP in predicting successful liberation from invasive ventilation.

**Funding:**

None.

**Research in context**

**Evidence before this study**

We searched PubMed from database inception to December 8, 2022, using the following search strategy: (“continuous positive airway pressure” [All Fields] OR “CPAP” [All Fields] OR “Pressure Support” [All Fields] OR “PSV” [All Fields] OR “T piece” [All Fields] OR “Ventilator liberation Protocol” [All Fields]) AND (“Extubation readiness test” [All Fields] OR “Spontaneous breathing test” [All Fields] OR “Breathing trial” [All Fields] OR “Spontaneous breathing trial” [All Fields] OR “SBT” [All Fields]) AND “child∗” [All Fields]. The search yielded 34 articles. Eight were articles related to ventilation and weaning in neonatal age group. Among the rest 26 articles, 20 were review articles, physiologic studies evaluating work of breathing during spontaneous breathing trial (SBT) and descriptive studies on weaning practice and predictors. There were 6 randomized trials of which 4 were studies that evaluated the effect of daily or protocolized SBT as a weaning strategy against usual care. Only 2 randomized controlled trials compared different methods of SBT for assessment of extubation readiness in children. One of them was a small trial in 36 children that compared pressure support with automatic tube compensation. The remaining trial was the only large study in children that compared pressure support against T-piece and showed similar rate of successful extubation between these two methods. However, there are no studies that tested pressure support against continuous positive airway pressure (CPAP) in children.

**Added value of this study**

To our knowledge, we present the first randomized controlled trial that compared pressure support with CPAP as SBT method for assessing extubation readiness in mechanically ventilated children. In this trial of 244 children undergoing invasive mechanical ventilation for >48 h, 2-h SBT with pressure support was found not inferior to 2-h SBT with CPAP in predicting successful liberation from invasive ventilation. The first SBT pass rate was also similar between these two methods. The need for non-invasive respiratory support after extubation was not greater with the use of pressure support SBT.

**Implications of all the available evidence**

Physiologic observational studies raised concerns regarding pressure support SBT underestimating post-extubation work of breathing that could lead to premature extubation and greater extubation failure in comparison to CPAP. The recent international clinical practice guidelines for pediatric ventilator liberation failed to arrive at a consensus for the preferred method of SBT between PS and CPAP due to lack of robust evidence. Our study results complement the prior randomized study that extubation failure rate is not higher when pressure support is used for SBT in clinical settings. Furthermore, the use of post-extubation non-invasive respiratory support, permitted in this study in line with the contemporary practice was not excessive in pressure support group, thus making a case for routine use of PS for SBT in children.

## Introduction

Invasive ventilation, although lifesaving, can be associated with significant short and long-term morbidities. Therefore, weaning from ventilatory support is imperative as soon as the underlying cause begins to resolve. The starting point and technique of weaning are less clearly defined and are often influenced by unit preferences and personal experiences.^1^^,^^2^ Successful extubation is a key endpoint of weaning, hence assessment for extubation readiness has received a lot of attention recently.^3^^,^^4^ Although the terms Spontaneous Breathing Trial (SBT) and Extubation Readiness Test (ERT) are used interchangeably in literature, SBT determines the ability to sustain spontaneous breathing with minimal support whereas ERT encompasses SBT as well as assessment of other elements (levels of sedation, neurological status, and risk of upper airway obstruction) required for successful extubation.^2^^,^^4^ Studies in mechanically ventilated adults have shown varied success rates of SBTs in reducing length of invasive ventilation, its complications, and healthcare costs.^5^, ^6^, ^7^, ^8^, ^9^, ^10^ In children, using daily SBTs for weaning reduced the risk of remaining on mechanical ventilation by 30% as compared to standard care.^11^ In a recent survey, about 86% of pediatric intensivists admitted to using SBT before extubation and up to 50% of them followed a protocolized SBT.^2^ Despite its widespread acceptance and use, the ideal technique and duration of SBT in children is still elusive. SBT in children is commonly performed using T-Piece, Continuous Positive Airway Pressure (CPAP), or CPAP with low level pressure support (PS) for a duration varying from 30 min to 2 h. The postulation regarding PS overcoming the resistance imposed by a narrow endotracheal tube while breathing spontaneously has been contested by recent reports as largely theoretical and clinically unfounded.^4^^,^^12^^,^^13^ Moreover, several physiologic studies in children suggested that PS could be an inferior method for SBT as it underestimated post-extubation work of breathing and thus could overestimate the rate of successful extubation. Khemani et al. reported that the pressure rate product, a surrogate for work of breathing was significantly lower on PS as compared to CPAP. PS and CPAP during SBT underestimated post-extubation effort by 126–147% and 17–25% respectively.^14^ Another randomized study shared similar physiologic observations that the pressure rate product was lowest for PS as compared to CPAP, T-piece, T-piece with heliox and post-extubation.^15^ Imposed work of breathing measured by tracheal manometry was also lower with PS when compared to CPAP.^16^

Despite physiologic concerns, for several reasons, PS for SBT is a highly preferred option among pediatric intensivists.^2^ Firstly, clinical studies in adults showed reassurance that PS was comparable to other methods in predicting successful extubation and reducing weaning duration.^9^^,^^10^^,^^17^ Secondly, PS being a less demanding method, may have higher SBT pass rate, thereby offsetting the gain achieved with CPAP in overall extubation outcomes.^18^^,^^19^ Lastly, the increasing availability and use of non-invasive ventilation after extubation could have also helped the cause in preferring PS for SBT.^2^ However the widespread use of PS for SBT is not backed by robust pediatric studies; data comparing PS with other methods of SBT in mechanically ventilated children are limited.^20^^,^^21^ The recent international guidelines for pediatric ventilator liberation suggested using either PS or CPAP during SBT but it was only a conditional recommendation due to very low certainty of evidence.^3^ To address this data gap, we hypothesized that PS, despite being physiologically inferior to CPAP, could result in comparable clinical outcomes. For pragmatic evaluation of this hypothesis, we compared PS with CPAP as SBT technique administered for 2 h in mechanically ventilated children.

## Methods

### Study design and participants

This was an open-label, prospective, parallel-group, randomised, non-inferiority trial conducted between December 2019 and August 2021 in a medical PICU of a tertiary care referral hospital in North India. The trial protocol was approved by the Institute Ethics Committee (Reference No. NK/5559/DM/848) and registered under Clinical Trial Registry of India (CTRI/2019/12/022328). Consecutive children aged between 1 month and 12 years with acute respiratory failure who received invasive mechanical ventilation for at least 48 h were screened for eligibility. Children who received mechanical ventilation for procedure/short term (≤48 h), for long duration (>4 weeks), and for end-of-life care or chronic neuromuscular disorders (requiring slow or customized weaning plan) were excluded. Eligible children were enrolled during the process of weaning, at the time point when they met the criteria for extubation readiness assessment (Ventilator rate ≤20, PIP ≤16, PEEP ≤6 and FiO_2_ ≤40%). Written informed consent was taken from parents/caregivers.

### Randomisation and masking

Patients were randomized in a 1:1 ratio to undergo a 2-h SBT with PS or CPAP using a computer-generated, block randomization with variable block sizes of 6, 8, 10 and 12 by a staff member not involved in the study. Allocation concealment was ensured using pre-sealed, sequentially numbered opaque envelopes that were opened only after obtaining a written informed consent. Masking could not be done.

### Procedure

Children allocated to CPAP group received a positive end expiratory pressure (PEEP) of 5–6 cm H_2_O on mechanical ventilator without any additional support. Instead, those in PS group received pressure support of 8 cm H_2_O in addition to PEEP of 5–6 cm H_2_O. Both groups were ventilated on conventional mechanical ventilators [Hamilton G5, Hamilton Medical AG, Switzerland] and received an inspired oxygen concentration [FiO_2_] between 21% and 40% based on their pre-SBT ventilator settings.

Children who successfully tolerated SBT for 2 h were extubated within 6 h of completion of SBT. Those who failed SBT were put back on the same ventilator settings that were on, prior to initiation of SBT. Failure to tolerate an SBT included increased work of breathing [intercostal or subcostal retractions, nasal flaring, tachypnoea, diaphoresis]; hypoxemia [decrease in pulse oximetry oxygen saturation (SpO_2_) below 94% or any increase in FiO_2_ requirement]; cardiovascular instability [tachycardia, bradycardia, or hypotension for age or a 20% change in heart rate or BP from baseline, presence of arrhythmia] or worsening sensorium [any decrease of GCS from baseline]. The treating team had the discretion to stop SBT at any point in time after documentation of reasons for stoppage. Peri-extubation dexamethasone was administered during weaning or SBT again at the discretion of the treating physician. Although the need for immediate post-extubation respiratory support with high flow nasal oxygen (HFNC) or non-invasive ventilation (NIV) was decided a-priori, the treating team was free to start respiratory support any time after extubation if clinically indicated. Extubation failure was defined as the need for re-intubation within 72 h of extubation.

Data with respect to basic demographic details, anthropometry, clinical features, presence of comorbidities, severity of illness, etiology, indications and details of invasive ventilation, use of sedo-analgesia and neuromuscular blockade, and other organ support care provided in PICU, were recorded on a predesigned proforma. PRISM-III score was documented using physiologic data during the initial 12 h of PICU care. MODS score was calculated as the sum of number of organ systems (Minimum score of 0 and Maximum score of 6); worst MODS score represented most concurrent organ dysfunctions during PICU stay.^22^ Duration of invasive ventilation, reasons for extubation failure, time to re-intubation, and details of post-extubation respiratory support were recorded. All children were followed up till hospital discharge and hospital outcomes including tracheostomy, discontinuation of care, and death were recorded.

### Outcomes

Primary outcome was successful liberation (free from invasive ventilation for at least 72 h) after first SBT. Secondary outcomes included first SBT pass rate, need for post-extubation respiratory support (HFNC or NIV), and length of PICU stay.

### Statistical analysis

The study was designed to test non inferiority of a 2-h SBT with PS when compared to a 2-h SBT with CPAP. Based on previous data that has reported 89% successful liberation after CPAP or T-piece in children receiving invasive ventilation, keeping a non-inferiority (NI) margin of 10%, 80% power and significance level (α) of 0.05, we calculated that 122 children in each group were required to determine that PS was non inferior to CPAP.^23^ We chose NI margin of 10% based on both clinical and statistical reasoning. Given that the current treatment (CPAP) as SBT method for successful weaning works 89% of the time, after thoughtful discussion with several clinicians, it was decided that if a response of at least 79% were achieved, the new treatment (PS) would be adopted. In addition, from a previous trial comparing CPAP versus T-piece for SBT in adults, the point estimate method of computing NI margin indicated a point difference in proportions (NI margin) of 10% (95% CI: −14.6% to 34.6%).^24^

Data analysis was done using STATA version 17.1 (StataCorp, College Station, Texas: StataCorp LLC) in the modified intention-to-treat population which included all children who underwent randomization except for 3 children who had extubation before the first SBT. Categorical variables are presented as absolute and relative frequencies. Continuous variables are summarized as medians and interquartile ranges (IQR) for non-normal distributions. Categorical variables were compared using the Chi-square or Fisher exact test. The Wilcoxon rank-sum test was used to compare non-parametric continuous variables. Effect sizes were measured using risk differences and differences between means with 95% confidence intervals for binary and continuous outcomes respectively. Time-to-event outcome (successful liberation from invasive ventilation) was analysed using Kaplan Meier curves and compared by log-rank test. As there was no death or discontinuation of care within 72 h post-extubation, there was no censoring.

### Role of funding source

Not applicable.

## Results

### Study participants

**Fig. 1:**
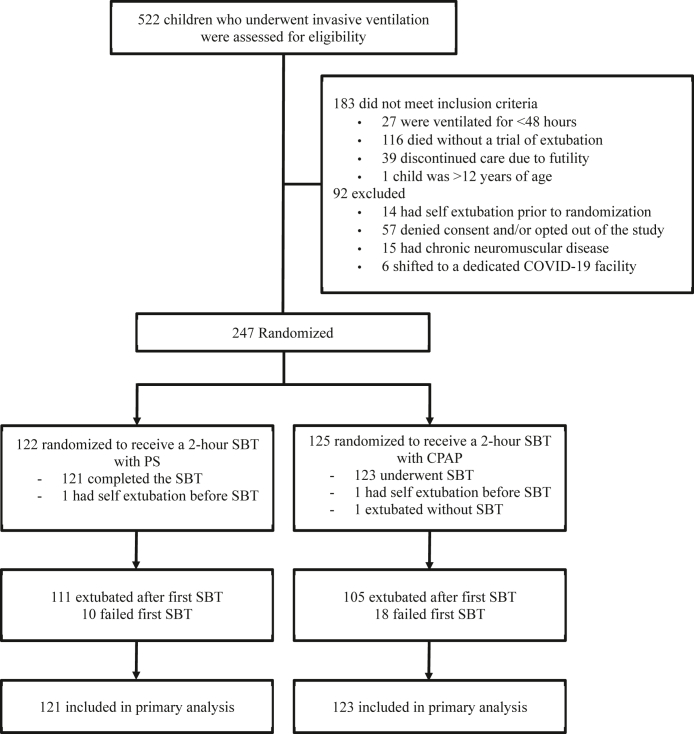
**Flow of participants in the study**.

A total of 522 children received mechanical ventilation during the study period, of whom 183 failed to meet the eligibility criteria (Deaths 116; Discontinuation of care—39; Invasive ventilation <48 h—27; Age >12 years—1). Furthermore, 92 children were excluded for various reasons (Accidental extubation prior to randomization—14, Chronic neuromuscular disease–15; Denied consent and/or opted out of the study by treating team—57, shifted to dedicated COVID-19 facility–6). Finally, 247 children were randomised, of which 244 completed the trial and were included in the primary analysis (123 received SBT with CPAP and 121 received SBT with PS). Three children did not undergo the trial intervention (2 in CPAP group and 1 in PS group). The study flow is depicted in Fig. 1.

The median (IQR) age of the study population was 24 (9, 84) months; about a third were infants younger than one year. The common reasons for PICU admission were bacterial sepsis [n = 50 (20.5%)], acute respiratory [n = 46 (19%)], and acute neurological [n = 45 (18.5%)] illnesses (Supplementary Table S1). About a quarter [n = 58; 23.8%] had comorbidities. Neurologic [n = 12, 5%] and hematologic/oncologic disorders [n = 12, 5%] were the common comorbidities (Supplementary Table S2). The two groups were comparable with respect to gender distribution, weight, severity of illness, organ dysfunction parameters (PRISM-III score, worst MODS score, worst oxygenation index (OI), lowest PaO_2_/FiO_2_ ratio, maximum fluid overload percentage), and use of peri-extubation dexamethasone. Median (IQR) duration of invasive ventilation before randomization [PS 4.5 (3, 7) versus CPAP 4.5 (3, 6) days] was comparable (Table 1). Both groups received sedation, analgesia and neuro-muscular blocking agents in similar proportion, dose, and duration (Supplementary Table S3).

**Table 1:** Baseline characteristics.

	PS group (n = 121)	CPAP group (n = 123)
Age (months) Median (IQR)	18 (9, 72)	24 (8, 84)
<1 year n (%)	38 (31.5)	41 (33.5)
1–5 years n (%)	49 (40.5)	45 (36.5)
6–12 years n (%)	34 (28)	37 (30)
Sex		
Male	73 (60)	65 (53)
Female	48 (40)	58 (47)
Weight (kg)	10 (8, 20)	10 (7, 20)
Weight <2 SD for age n (%)	22 (18)	27 (22)
Reason for admission		
Respiratory diseases	23 (19)	23 (19)
Central nervous system diseases	19 (16)	26 (21)
Bacterial sepsis	25 (21)	30 (24.5)
Tropical Infections	8 (6.5)	14 (11.5)
Acute neuromuscular diseases	10 (8)	10 (8)
Metabolic disorders	9 (7.5)	7 (5.5)
Cardio-vascular diseases	6 (5)	1 (1)
Hematologic/Oncologic diseases	2 (1.5)	2 (1.5)
Hepatobiliary and gastro-intestinal diseases	4 (3)	2 (1.5)
Others	15 (12.5)	8 (6.5)
Comorbidities	30 (25)	28 (23)
PRISM-III Score	14 (11, 18)	15 (10, 18)
Worst MODS score	3 (1, 4)	3 (1, 4)
Worst oxygenation index	3.5 (2.5, 5.7)	3.6 (2.4, 5.1)
Lowest PaO_2_/FiO_2_ ratio	300 (210, 391)	273 (212, 378)
Maximum fluid overload (FO %)	8.7 (4.5, 15)	8 (4.1, 12.4)
Sedation and analgesia use n (%)		
Midazolam	113 (93)	122 (99)
Fentanyl	114 (94)	116 (94)
Dexmedetomidine	8 (7)	13 (11)
Ventilation parameters (Highest/worst value)		
PIP cm of H_2_O	18 (16, 20)	18 (16, 21)
PEEP cm of H_2_O	6 (5, 7)	6 (5, 8)
MAP cm of H_2_O	10 (9, 12)	10 (9, 12)
RR (per minute)	25 (25, 30)	25 (25, 30)
FiO_2_ (%)	40 (30, 40)	40 (30, 45)
Ventilation parameters at randomization		
PIP cm of H_2_O	15 (15, 16)	15 (15, 16)
PEEP cm of H_2_O	5 (5, 6)	5 (5, 6)
MAP cm of H_2_O	9 (8, 9)	8 (8, 9)
Tidal volume (mL/kg)	10 (8, 10)	10 (8, 12)
FiO_2_ (%)	30 (25, 30)	30 (25, 35)
Length of invasive ventilation prior to first SBT (days)	4.5 (3, 7)	4.5 (3, 6)
Peri-extubation dexamethasone		
Used in n (%)	102 (84)	98 (80)
Duration between first dose and extubation (hours)	18 (12, 24)	12 (12, 24)
Dose (mg/kg)	0.15 (0.15, 0.2)	0.15 (0.15, 0.25)
Total number of doses	4 (4, 6)	4 (4, 6)

CPAP; Continuous positive airway pressure, PS; Pressure support, SBT; Spontaneous breathing trial, MODS; Multi-organ dysfunction syndrome, PaO_2_; Partial pressure of arterial oxygen, FiO_2_; Percentage of fractional inspired oxygen, PaCO_2_; Partial pressure of arterial carbon dioxide, PIP; Peak inspiratory pressure, PEEP; Positive end expiratory pressure, MAP; Mean airway pressure, RR; respiratory rate.

Values are expressed in median (interquartile range) or numbers (%).

### Primary outcome

One hundred and eleven children (91.7%) in PS and 105 (85.4%) in CPAP group were extubated after first SBT. Among them, successful liberation from invasive ventilation occurred in 97 (80.2%) in the PS and in 93 (75.6%) in the CPAP group. The mean difference was 4.6% [95% CI, −5.8% (favouring CPAP) to 15% (favouring PS); the lower limit of 95% CI did not exceed the predefined NI margin] (Table 2).

**Table 2:** Outcome measures.

	PS group (n = 121)	CPAP group (n = 123)	Difference, PS minus CPAP (95% CI)	p value
**Primary outcome**				
Successful extubation after first SBT n (%)	97 (80.2)	93 (75.6)	4.6 (−5.8, 15)	0.39
**Secondary outcomes**				
First SBT pass n (%)	111 (91.7)	105 (85.4)	6.3 (−1.6, 14.3)	0.12
Interval between first SBT and extubation (hours)	2 (1.5, 4)	2 (1, 3.5)	0.24 (−0.13, 0.62)	0.15
Reintubation within 72 h n (%)	14 (11.6)	12 (9.8)	1.8 (−5.9, 9.6)	0.65
Time to re-intubation (hours)	24 (15.5, 36)	18 (6.75, 58)	−6.2 (−23.2, 10.8)	0.98
Need for post extubation respiratory support n (%)	52 (43)	49 (40)	3.1 (−9.2, 15.5)	0.62
Reason for post-extubation respiratory support n (%)				
Decided a priori	23 (19)	15 (12)	6.8 (−2.3, 15.9)	0.14
Increased work of breathing	23 (19)	23 (19)	0.3 (−9.5, 10.1)	
Upper airway obstruction	3 (2.5)	3 (2.5)	0 (−3.8, 3.9)	
Cardiac dysfunction	1 (1)	2 (1.5)	−0.8 (−3.6, 2)	
Neurological/neuromuscular reasons	2 (1.5)	6 (5)	−3.2 (−7.7, 1.2)	
Successful extubation after 2nd SBT n (%)	10 (8.3)	17 (13.8)	−5.5 (−13.4, 2.3)	0.17
All reintubations at any point in time n (%)	17 (14)	18 (14.6)	−0.6 (−9.4, 8.2)	0.90
Tracheostomy n (%)	6 (5)	3 (2.5)	2.5 (−2.2, 7.3)	0.30
Length of invasive ventilation (days)	5.5 (3, 7)	5 (3.5, 7)	0.1 (−1.3, 1.5)	0.96
Length of PICU stay (days)	9 (6, 15)	8 (5.5, 13)	1 (−1, 3)	0.48
Length of hospital stay (days)	16 (10, 25)	16 (10, 22)	1.9 (−0.9, 4.7)	0.56
Unfavourable outcome n (%)	0 (0)	4 (3.3)	−3.3 (−6.4, −0.1)	
Death	0 (0)	1 (0.8)		
Discontinued care	0 (0)	3 (2.5)		

Values are expressed in numbers (%) or median (interquartile range).

### Secondary outcome

**Fig. 2:**
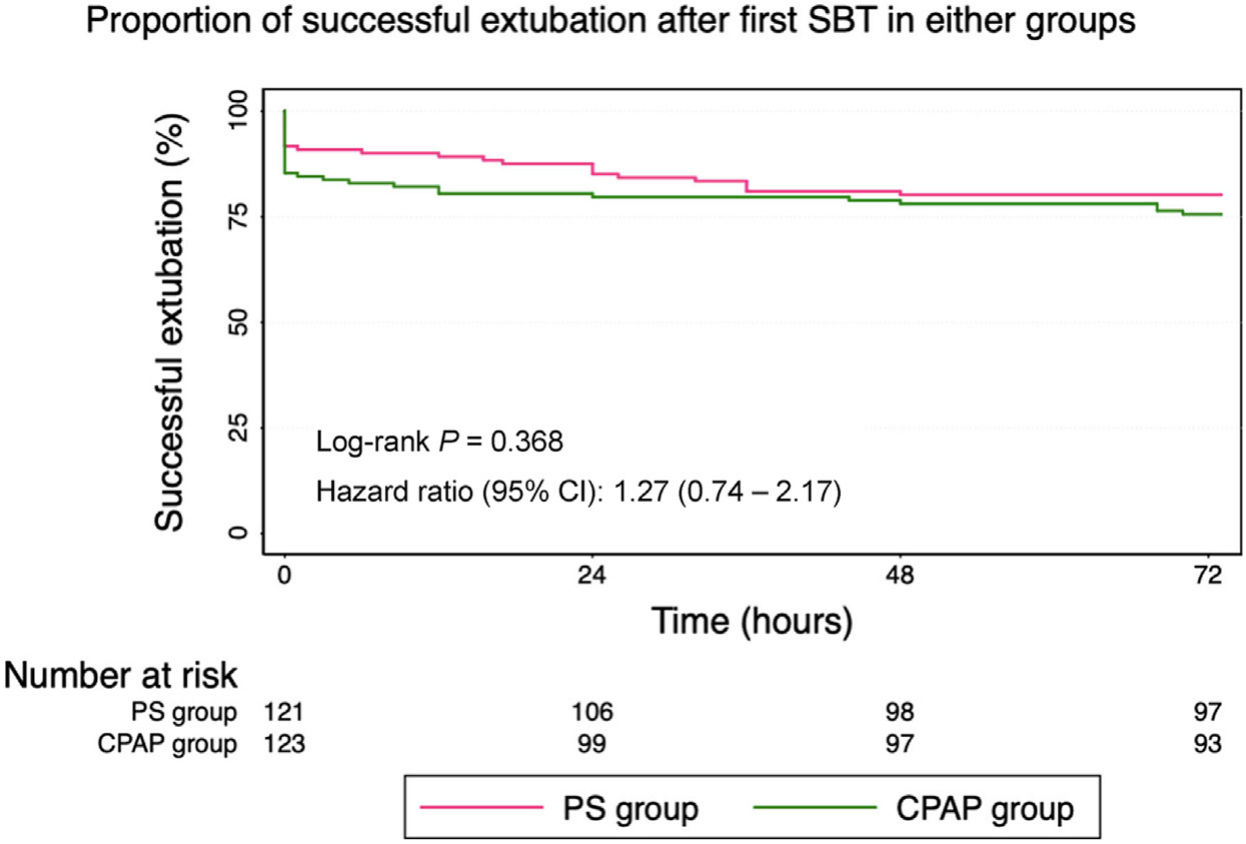
**Time to event data of successful extubation**.

First SBT pass rate between PS and CPAP groups was similar [111 (91.7%) versus 105 (85.4%); difference 6.3%; 95% CI (−1.6, 14.3); p = 0.12]. Twenty six (10.7%) children needed reintubation within 72 h; overall reintubation rate [14 (11.6%) versus 12 (9.8%); difference 1.8%; 95% CI (−5.9, 9.6); p = 0.65] and the adjusted reintubation rate after excluding cases [n = 10, 4%] with upper airway obstruction [9 (7.4%) versus 7 (5.7%); difference 1.7%; 95% CI (−4.5, 8); p = 0.65] were comparable for the PS versus CPAP group respectively. The median (IQR) time to reintubation was also similar among PS and CPAP groups [24 (15.5, 36) versus 18 (6.75, 58) hours; difference −6.2; 95% CI (−23.2, 10.8) hours; p = 0.98]. The Kaplan–Meier curves showed no significant difference in the rates to successful liberation from invasive ventilation [hazard ratio (95% CI)—1.27 (0.74, 2.17); p = 0.37] (Fig. 2).

Post-extubation respiratory support (HFNC or NIV) was used in 101 (41.4%) children; decided a-priori before extubation in 38 (15.6%) and used as rescue measure in 63 (25.8%). The rate of rescue support [29 (24%) in PS group and 34 (27.6%) in CPAP group; difference −3.6%; 95% CI (−14.6, 7.3); p = 0.51] was similar. Six (5%) children in the PS group and 3 (2.5%) in the CPAP group required tracheostomy [difference 2.5%; 95% CI (−2.2, 7.3); p = 0.30)]. The median (IQR) length of PICU stay was 9 (6, 15) days in the PS group and 8 (5.5, 13) days in the CPAP group [mean difference 1; 95% CI (−1, 3) days; p = 0.48]. The median (IQR) length of hospital stay was 16 (10, 25) days in PS group and 16 (10, 22) days in CPAP group [mean difference 1.9; 95% CI (−0.9 to 4.7) days; p–0.56]. Four (1.6%) children, all in CPAP group had unfavourable outcomes (1 died and 3 discontinued care) (Supplemental Fig. S1).

## Discussion

This study compared the weaning outcomes between PS and CPAP when used for SBT in mechanically ventilated children. PS was as good as CPAP with respect to rates of successful extubation, first SBT pass rate, time to reintubation, and need for post extubation respiratory support. These findings are in concurrence with randomized experiments done in adults that have reported comparable or even superior weaning outcomes with SBT using PS versus CPAP or T-piece.^7^^,^^9^ Despite a physiologic metanalysis raising concerns for the use of PS for SBT in adults, large clinical trials and meta-analysis of clinical studies have found PS to be a suitable method.^7^^,^^9^^,^^10^^,^^18^^,^^25^^,^^26^ Most of them reported greater SBT pass rate with PS and observed that patients were more likely to be extubated successfully with PS in comparison to T-piece SBT. The success rate was attributed to the less demanding nature of PS during SBT that allowed critically ill patients to sustain breathing more effectively.^9^

SBTs assess the ability of a patient to sustain spontaneous breathing post extubation. An ideal SBT should mimic the work of breathing that occurs without the ventilatory support and endotracheal tube (ETT) in situ.^4^ The assessment of extubation readiness in children however differs from adults as the ETTs are smaller and variable in size. Unlike adults, there is a component of resistive work of breathing that needs to be overcome in children. The proponents of PS for SBT in children therefore argue for addition of low levels of pressure support adjusted for the internal diameter of the ETT to overcome the tube resistance.^13^ However, the accurate level of pressure required to overcome this resistive work in children is not clearly defined and is largely arbitrary (5–10 cm H_2_O). In the current study, we used a pressure support of 8 cm H_2_O in PS group irrespective of ETT diameter. The opponents of PS argue that resistance is not solely determined by tube diameter but is a function of flow in addition to diameter. It has been shown that, at usual peak inspiratory flow, the resistance generated even in smaller ETTs was low and clinically insignificant.^12^ Khemani et al. demonstrated that use of pressure support underestimated post extubation work of breathing by 126–147% while CPAP underestimated it only by 17–25%, thus flagging the concern for the use of PS in SBT.^14^ Despite these conflicting physiologic arguments, PS continues to be the most popular method for SBT in PICU. An international survey of ventilation liberation practice showed that about 82% pediatric intensivists preferred PS over CPAP for SBT in children.^2^ The recent clinical practice guidelines however failed to arrive at a consensus for the preferred method of SBT due to lack of robust evidence.^3^ The only previous randomized controlled trial in children by Farias et al. comparing 2-h SBT using PS of 10 cm H_2_O versus T-piece showed no significant difference in extubation failure between the two methods.^20^ Our results complement the findings of this trial in that the extubation failure rates are not higher with PS SBT in clinical settings. Our study differs from the previous RCT on two accounts. Firstly, we used CPAP instead of T-piece as comparator owing to the physiological evidence that CPAP is similar to T-piece in mimicking post-extubation work of breathing. Also, administering CPAP avoids disconnection from ventilator that has advantages in ensuring humidification, monitoring of expiratory volume and functioning of alarms during SBT.^27^ Secondly, up to 40% of children in our study received post extubation NIV, both as decided a priori and as a rescue measure for increased work of breathing any time after extubation. We allowed NIV in both the groups at the clinician's discretion as the practice is pragmatic and has become the standard of care in most units. Use of rescue NIV was not excessive in PS group as compared to CPAP group, further supporting the use of PS for SBT in children.

The optimal duration of SBT is another contentious issue.^28^^,^^29^ Studies in adults have shown that a 30-min SBT was equivalent to 120-min SBT in assessing extubation readiness with no difference in extubation failure rate.^9^ However, the SBT durations tended to be longer in most pediatric studies.^20^^,^^21^ A recent secondary analysis of a clinical trial in Pediatric Acute Respiratory Distress Syndrome (PARDS) concluded that a 30-min SBT may be too short in children recovering from PARDS as about 40% who passed SBT at 30 min went on to fail the SBT between 30 and 120-min.^30^ We chose 2 h duration for SBT as it has been used most frequently and is the more strenuous. Our study however may not have answered the question of optimal duration of SBT. A shorter SBT with PS may be worth evaluating if that helps in improving weaning outcomes.

Our study has several strengths. To the best of our knowledge this is the first RCT comparing PS with CPAP for assessing extubation readiness in children. The study was pragmatic as it included all ventilated children irrespective of underlying illness. No changes were made in weaning protocol till the decision of SBT was made by the treating team. There were no restrictions in instituting post extubation NIV support. However, a few limitations need mention. The study was not blinded as blinding during SBT would have hindered the monitoring of ventilator variables. We believe that the disadvantages of open label design were possibly minimized by the objective nature of study outcomes. The predefined NI margin in this study was based on statistical reasoning from an adult trial due to paucity of historical trials comparing CPAP with T-piece in children. A three arm trial including T-piece could have allowed some within-trial validation of the choice of NI margin, however, it may not have added clinical relevance as T-piece is less commonly used in children for SBT in contemporary practice.^2^^,^^3^

To conclude, a 2-h SBT with PS is not inferior to 2-h SBT with CPAP in predicting successful liberation from invasive mechanical ventilation in children. We propose a larger trial to test the superiority of PS over CPAP for SBT in predicting weaning outcomes in children.

## Contributors

This study was conceived and designed by KN and CRV. Trial management and conduct were done by MJ, SKA, AB and KN. Patient enrolment and data collection were done by CRV. CRV and KN developed the statistical analysis plan. All authors had access to the raw data and CRV undertook statistical analysis. The manuscript was drafted by CRV and KN. All authors reviewed, contributed to revisions, and approved the final version of the manuscript. CRV and KN have directly accessed and verified the underlying data reported in the manuscript.

## Data sharing statement

Data sharing requests will be considered from research groups that submit a research proposal and an appropriate statistical analysis plan for individual participant data meta-analysis. Requests for de-identified data up to 5 years following article publication should be directed to ny.karthi@gmail.com.

## Declaration of interests

All authors declare no competing interests.

## Appendix A Supplementary data

Supplementary material

CTRI registration_Protocol_15-May
