# Supplementary material for: Pressure support versus continuous positive airway pressure for predicting successful liberation from invasive ventilation in children: an open label, randomized non-inferiority trial
Source: Lancet Reg Health Southeast Asia. 2023 May 29;14:100219. doi: 10.1016/j.lansea.2023.100219 (PMC10363498; doi:10.1016/j.lansea.2023.100219)

**Supplementary content:**

**Supplementary table S1: Details of diagnoses**

| **Diagnosis** | **PS group (n = 121)** | **CPAP group (n = 123)** |
| --- | --- | --- |
| Respiratory   - Pneumonia - Acute bronchiolitis - Hydatid cyst of lung - Bacterial tracheitis - Pneumothorax - Airway foreign body aspiration - COVID-19 pneumonia - Tracheo-oesophageal fistula - Airway injury - Status asthmaticus - Chylothorax | 12  2  0  0  1  4  0  0  3  0  1 | 10  3  2  1  0  3  1  1  1  1  0 |
| Central Nervous System diseases   - Acute meningoencephalitis - Acute meningitis - Intracranial bleed - Acute afebrile encephalopathy - Febrile seizures - Status epilepticus - Reye like illness - Cerebral vein thrombosis | 3  3  5  1  1  3  1  2 | 11  3  7  2  0  2  0  1 |
| Bacterial sepsis   - Septic shock - Toxic shock syndrome - Disseminated staphylococcal sepsis - Necrotising fasciitis - Purpura fulminans - Steven Johnson syndrome with secondary sepsis - Febrile neutropenia - Deep neck space infection | 13  3  3  1  1  1  0  3 | 17  2  9  1  0  0  1  0 |
| Tropical infections   - Dengue - Scrub typhus - Others | 2  3  3 | 4  7  3 |
| Cardiovascular diseases   - Congestive cardiac failure - Massive pericardial effusion - Fulminant myocarditis - Low cardiac output state | 2  1  2  1 | 1  0  0  0 |
| Acute neuromuscular diseases   - Neuroparalytic snake envenomation - Generalized tetanus - Compressive myelopathy | 9  1  0 | 9  0  1 |
| Hematologic / Oncologic diseases   - Tumour lysis syndrome - Severe Anemia - Rhabdomyosarcoma - Lung Mass | 0  1  1  0 | 1  0  0  1 |
| Hepatobiliary and gastro-intestinal diseases   - Hepatic encephalopathy - Acute severe pancreatitis - Intestinal perforation | 4  0  0 | 0  1  1 |
| Metabolic disorders   - Diabetic ketoacidosis - Inborn error of metabolism | 3  6 | 3  4 |
| Others   - Poisoning - Electrocution - Polytrauma - Multisystem inflammatory syndrome in children - Drowning | 6  0  1  8  0 | 2  1  1  3  1 |

**Supplementary table S2: Details of comorbidities**

| **Comorbidities** | **PS group (n = 121)** | **CPAP group (n = 123)** |
| --- | --- | --- |
| Cardiovascular  Congenital Heart Disease  Dilated cardiomyopathy | 2  1 | 0  1 |
| Respiratory  Cystic fibrosis  Bronchial asthma  Tracheo-oesophageal fistula | 0  0  0 | 1  1  1 |
| Neurological  Epilepsy  Infantile tremor Syndrome  Intra-cranial cavernoma  Arachnoid cyst | 2  3  1  0 | 2  3  0  1 |
| Hematologic  Haemophilia A  Leukemia  Aplastic anemia  Infantile pyknocytosis | 1  2  1  0 | 0  3  0  1 |
| Genetic syndromes  ROHHAD syndrome  Turner syndrome  Down's syndrome | 2  1  1 | 0  0  0 |
| Endocrine  Type I Diabetes Mellitus  Cushing syndrome | 3  1 | 3  0 |
| Renal  Nephrotic syndrome  Chronic kidney disease | 2  0 | 0  1 |
| Tumours  Craniopharyngioma  Rhabdomyosarcoma  Chest mass | 0  1  0 | 1  1  1 |
| Others  Inborn error of metabolism  Neonatal cholestasis | 6  0 | 6  1 |
| **Total** | **30** | **28** |

**Supplementary table S3: Details of sedation, analgesia and neuromuscular blockade**

|  | **PS group (n = 121)** | **CPAP group (n = 123)** |
| --- | --- | --- |
| Midazolam  Used in n(%)  Dose (mcg/Kg/Min) (IQR)  Duration (in hours) (IQR) | 113 (93)  2 (2 – 4)  90 (60 – 132) | 122 (99)  2 (2 – 4)  84 (60 – 132) |
| Lorazepam (boluses)  Used in n(%)  Dose (mg/Kg)  Number of doses (IQR) | 9 (7.5)  0.1  20 (18 – 78) | 5 (4)  0.1  26 (20 – 30) |
| Dexmedetomidine  Used in n(%)  Dose (mcg/kg/Hour)  Duration (in hours) (IQR) | 8 (7)  0.3 (0.25 – 0.3)  78 (54 – 120) | 13 (11)  0.4 (0.25 – 0.5)  48 (36 – 72) |
| Fentanyl  Used in n(%)  Dose (mcg/Kg/Hour)  Duration (in hours) (IQR) | 114 (94)  2  98 (60 – 144) | 116 (94)  2  96 (57 – 144) |
| Ketamine  Used in n(%)  Dose (mg/Kg/Hour)  Duration (in hours) (IQR) | 5 (4)  1  24 (17 – 48) | 9 (7.5)  1  48 (36 – 84) |
| Morphine  Used in n(%)  Dose (mcg/Kg/Hour)  Duration (in hours) (IQR) | 14 (11.5)  20  72 (36 – 156) | 9 (7.5)  20 (20 – 30)  108 (96 – 192) |
| Vecuronium  Used in n(%)  Dose (mg/Kg/Hour)  Duration (in hours) (IQR) | 1 (1)  0.1  144 | 5 (4)  0.1  48 (12 – 60) |
| Atracurium  Used in n(%)  Dose (mg/Kg/Hour)  Duration (in hours) (IQR) | 1 (1)  0.1  60 | 0 (0)  0  0 |

**Supplementary table S4: Monitoring details during SBT**

|  | **PS group**  **(n = 121)** | **CPAP group**  **(n = 123)** |
| --- | --- | --- |
| Highest RR | 28 (23 – 32) | 29 (24 – 34) |
| P/F ratio | 413 (330 – 493) | 430 (337 – 500) |
| PaCO_2_ | 38 (34 – 44) | 40 (35 – 46) |

*RR – Respiratory rate, PaCO_2_ – Partial pressure of CO_2_ in arterial blood, P/F ratio – PaO_2_ / FiO_2_ ratio*

**Supplementary table S5: Reasons for reintubation**

|  | **PS group**  **(n = 14)** | **CPAP group**  **(n = 12)** | **Difference, PS minus CPAP (95% CI)** |
| --- | --- | --- | --- |
| Upper airway tract obstruction | 5 (35.6) | 5 (41.7) | -6 (-43.5, 31.6) |
| Lung collapse / parenchymal disease | 6 (42.9) | 4 (33.3) | 20.6 (-11.6, 52.9) |
| Fluid overload | 0 (0) | 2 (16.7) | -16.7 (-37.8, 4.4) |
| Cardiac dysfunction / pulmonary edema | 2 (14.3) | 0 (0) | 14.3 (-4, 32.6) |
| Neurologic abnormality | 1 (7.2) | 1 (8.4) | -1.2 (-21.8, 19.5) |

**Supplementary Figure S1: Study flow and participants outcome**


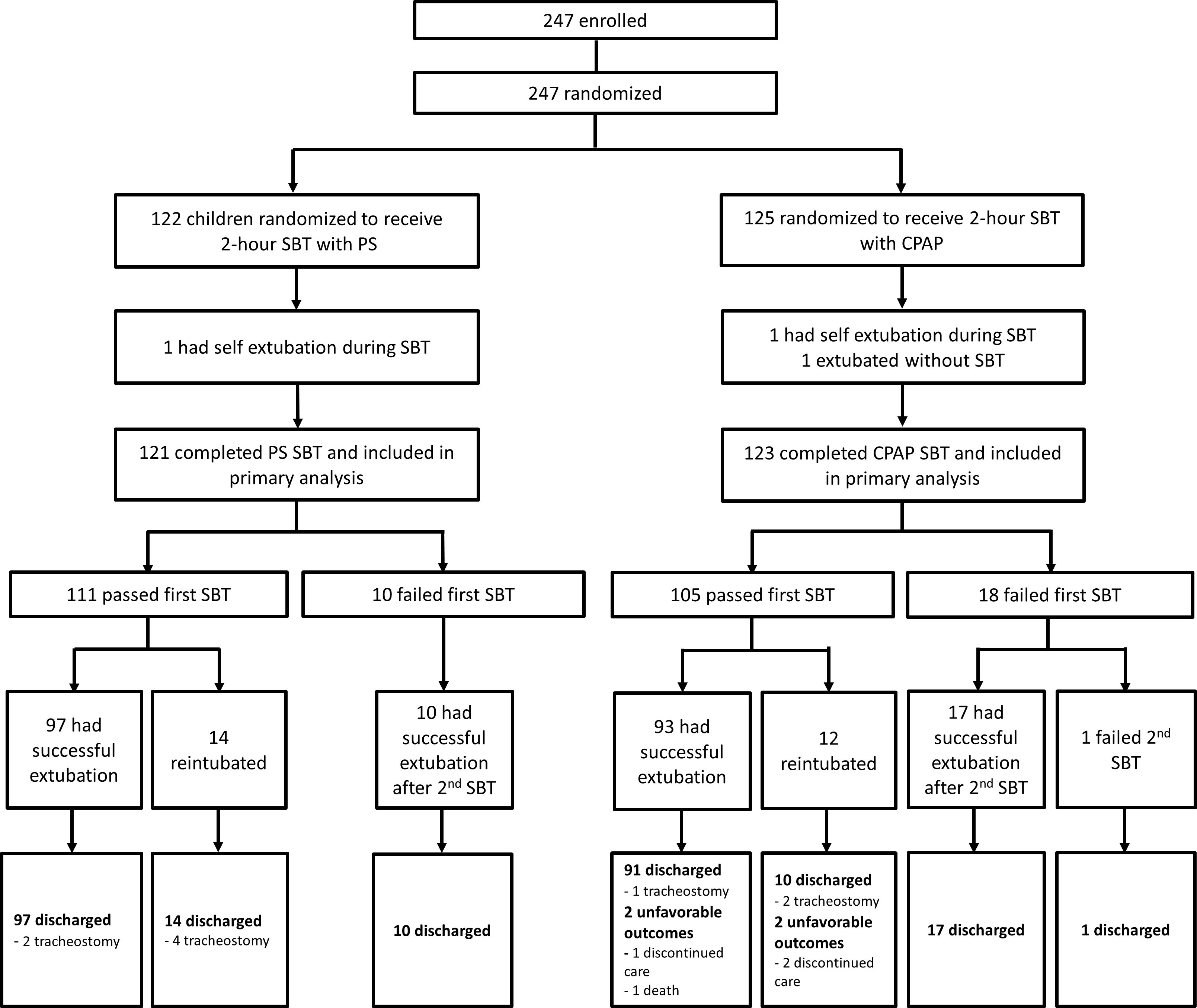

Supplement: Supplementary material [file mmc1.docx]
