# Supplementary material for: Pressure support versus continuous positive airway pressure for predicting successful liberation from invasive ventilation in children: an open label, randomized non-inferiority trial
Source: Lancet Reg Health Southeast Asia. 2023 May 29;14:100219. doi: 10.1016/j.lansea.2023.100219 (PMC10363498; doi:10.1016/j.lansea.2023.100219)
Supplement: CTRI registration_Protocol_15-May [file mmc2.pdf]

Clinical Trial Details (PDF Generation Date :- Fri, 12 May 2023 07:21:36 GMT)

**CTRI Number**	CTRI/2019/12/022328 [Registered on: 12/12/2019] - **Trial Registered Prospectively**
**Last Modified On**	11/12/2019
**Post Graduate Thesis**	Yes
**Type of Trial**	Interventional
**Type of Study**	Other (Specify) [Ventilation weaning strategy]
**Study Design**	Randomized, Parallel Group, Active Controlled Trial
**Public Title of Study**	A RANDOMISED CONTROLLED TRIAL COMPARING TWO METHODS OF EXTUBATOON READINESS TRIAL (CPAP AND PRESSURE SUPPORT) IN MECHANICALLY VENTILATED CHILDREN
**Scientific Title of Study**	PRESSURE SUPPORT VERSUS CPAP FOR EXTUBATION READINESS IN CHILDREN RECEIVING MECHANICAL VENTILATION: A RANDOMIZED CONTROLLED TRIAL

Secondary IDs if Any

Secondary ID	Identifier
NIL	NIL

Details of Principal Investigator or overall Trial Coordinator (multi-center study)

Details of Principal Investigator	
**Name**	Karthi N
**Designation**	Assistant Professor
**Affiliation**	Postgraduate Institute of Medical Education and Research
**Address**	Department of Paediatrics Advanced Paediatric Center PGIMER, Sector 12 Chandigarh<br>Chandigarh<br>CHANDIGARH<br>160012<br>India
**Phone**	9814376716
**Fax**	
**Email**	ny.karthi@gmail.com

Details Contact Person (Scientific Query)

Details Contact Person (Scientific Query)	
**Name**	Karthi N
**Designation**	Assistant Professor
**Affiliation**	Postgraduate Institute of Medical Education and Research
**Address**	Department of Paediatrics Advanced Paediatric Center PGIMER, Sector 12 Chandigarh<br>Chandigarh<br>CHANDIGARH<br>160012<br>India
**Phone**	9814376716
**Fax**	
**Email**	ny.karthi@gmail.com

Details Contact Person (Public Query)

Details Contact Person (Public Query)	
**Name**	Vishwa CR
**Designation**	Senior Resident
**Affiliation**	Postgraduate Institute of Medical Education and Research
**Address**	Department of Paediatrics Advanced Paediatric Center PGIMER, Sector 12 Chandigarh<br>Chandigarh<br>CHANDIGARH<br>160012<br>India

**Phone**	8800517848
**Fax**	
**Email**	drvishwacr@gmail.com

Source of Monetary or Material Support

Source of Monetary or Material Support
> PGIMER, Chandigarh

Primary Sponsor

Primary Sponsor Details	
**Name**	PGIMER Chandigarh
**Address**	PGIMER, Sector 12, Chandigarh, 160012
**Type of Sponsor**	Research institution and hospital

Details of Secondary Sponsor

Name	Address
NIL	NIL

Countries of Recruitment

List of Countries
India

Sites of Study

Name of Principal Investigator	Name of Site	Site Address	Phone/Fax/Email
Vishwa CR	Postgraduate Institute of Medical Sciences and Research, Chandigarh	Department of Paediatric Critical Care, Paediatric Department Office, 3rd Floor, Advanced Paediatric Center, PGIMER Chandigarh<br>CHANDIGARH	8800517848<br><br>drvishwacr@gmail.com

Details of Ethics Committee

Name of Committee	Approval Status	Date of Approval	Is Independent Ethics Committee?
INSTITUTE ETHICS COMMITTEE	Approved	14/11/2019	No

Regulatory Clearance Status from DCGI

Status	Date
Not Applicable	No Date Specified

Health Condition / Problems Studied

Health Type	Condition
Patients	Diseases of the respiratory system

Intervention / Comparator Agent

Type	Name	Details
Intervention	Pressure Support	A mode used for extubation readiness test - 2 hours
Comparator Agent	CPAP	A mode used for extubation readiness test - 2 hours

Inclusion Criteria

Inclusion Criteria	
**Age From**	1.00 Month(s)
**Age To**	12.00 Year(s)
**Gender**	Both
**Details**	1. Children aged 12 years or younger and receiving mechanical ventilation at least for 48 hours 2. Informed written consent

Exclusion Criteria

Exclusion Criteria	
**Details**	1. Procedural or short term intubation<br>2. End of life care<br>3. Neuromuscular disorders requiring slow or customised weaning plan<br>4. Prolonged mechanical ventilation >4 weeks

**Method of Generating Random Sequence**	Computer generated randomization
**Method of Concealment**	Sequentially numbered, sealed, opaque envelopes
**Blinding/Masking**	Outcome Assessor Blinded

Primary Outcome

Outcome	Timepoints
Successful extubation after first extubation readiness test	72 hours post extubation

Secondary Outcome

Outcome	Timepoints
Rate of re-intubation	72 hours post extubation
Need for post extubation NIV	72 hours post extubation
Ventilation free days	Till discharge from PICU
Length of PICU stay	Till discharge from PICU

**Target Sample Size**	**Total Sample Size=**244<br>**Sample Size from India=**244<br>**Final Enrollment numbers achieved (Total)=**Applicable only for Completed/Terminated trials<br>**Final Enrollment numbers achieved (India)=**Applicable only for Completed/Terminated trials
**Phase of Trial**	N/A
**Date of First Enrollment (India)**	12/12/2019
**Date of First Enrollment (Global)**	No Date Specified
**Estimated Duration of Trial**	**Years=**2<br>**Months=**2<br>**Days=**0
**Recruitment Status of Trial (Global)**	Not Applicable
**Recruitment Status of Trial (India)**	Not Yet Recruiting
**Publication Details**	Nil
**Brief Summary**	This is a single centre randomised controlled study at a tertiary centre in North India. Children will be randomised to CPAP or Pressure support for extubation readiness trial. The primary outcome is rate of successful extubation after first ERT. Secondary outcomes include rate of re-intubation, need for post extubation NIV, ventilation free days and length of PICU stay.
